# Considerations in computer-aided design for inlay cranioplasty: technical note

**DOI:** 10.1007/s10006-017-0668-4

**Published:** 2018-01-08

**Authors:** Erik Nout, Maurice Y. Mommaerts

**Affiliations:** 1 European Face Centre, Universitair Ziekenhuis Brussel, VUB, Brussels, Belgium; 2 Division of Oro-Maxillo-Facial Surgery, GH Elisabeth-Tweesteden, Tilburg, The Netherlands

**Keywords:** Cranium, Implant, Computer-aided design

## Abstract

**Context:**

Cranioplasty is a frequently performed procedure that uses a variety of reconstruction materials and techniques. In this technical note, we present refinements of computer-aided design–computer-aided manufacturing inlay cranioplasty.

**Objective, design, and setting:**

In an attempt to decrease complications related to polyether-ether-ketone (PEEK) cranioplasty, we gradually made changes to implant design and cranioplasty techniques. These changes include under-contouring of the implant and the use of segmented plates for large defects, microplate fixation for small temporal defects, temporal shell implants to reconstruct the temporalis muscle, and perforations to facilitate the drainage of blood and cerebrospinal fluid and serve as fixation points.

**Results:**

From June 2016 to June 2017, 18 patients underwent cranioplasty, and a total of 31 PEEK and titanium implants were inserted. All implants were successful.

**Conclusions:**

These changes to implant design and cranioplasty techniques facilitate the insertion and fixation of patient-specific cranial implants and improve esthetic outcomes.

## Introduction

Large skull defects (> 25 cm^2^) are reconstructed to protect the underlying brain, shield the brain from infection, and correct esthetic deformities [1]. However, it is unclear whether reconstruction is appropriate for the treatment of “syndrome of the trephined” or to decrease seizure activity [2, 3]. When grafting of the original bone flap fails because of infection and/or resorption, reconstruction can be performed using autologous split-calvarial grafts, poly-methyl-methacrylate (PMMA) that is manually preformed or computer numerical control (CNC)-milled, calcium phosphate (hydroxyapatite), titanium alloy sheets, titanium mesh, three-dimensional (3D)-printed Ti–6Al–4V titanium alloy, or CNC-milled polyether-ether-ketone (PEEK).

The 3D printing and CNC milling of the reconstruction plate are categorized as class I computer-aided design and computer-aided manufacturing (CAD-CAM) [4]. Plate design requires close attention to cosmesis, flap dissection (access), and prevention of epidural fluid collection and wound dehiscence. The outer contour is reproduced by mirroring in medical imaging processing software or by de novo design. Holes are required for fluid exchange, tissue integration, osteosynthesis, dura re-expansion, and temporalis muscle resuspension. However, little attention has been given to the design details.

Although we support the use of PEEK plates for reconstruction of calvarial defects, our group has observed numerous complications when using this material [5]. Therefore, we have gradually changed the implant design to decrease the incidence of complications, besides looking for alternatives with increased osseointegration properties (ceramic-titanium). The objective of this technical note is to present design refinements of CAD-CAM inlay cranioplasty in general.

## Materials and methods

**Fig. 1:**
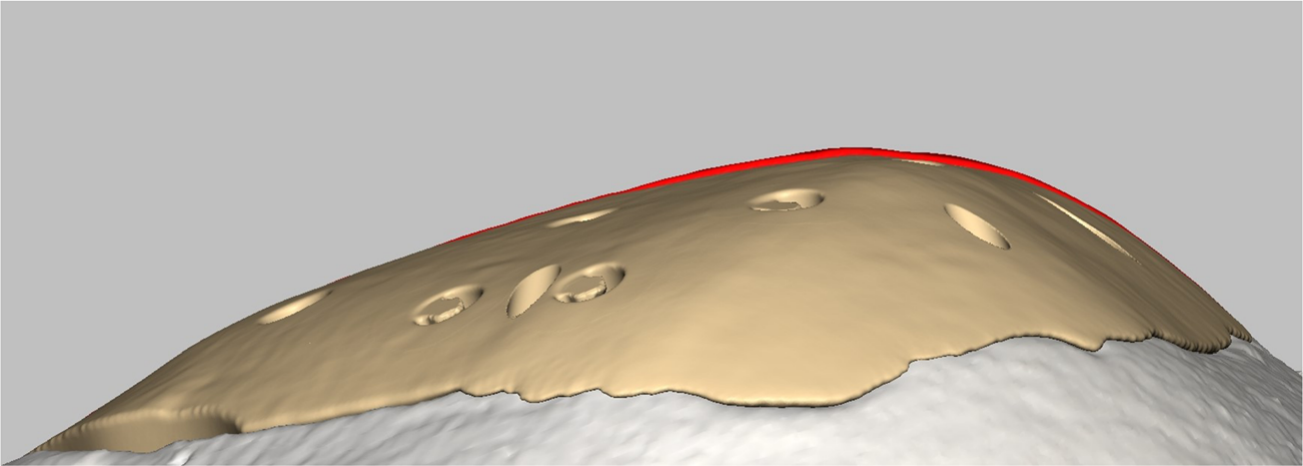
The mirrored contour is slightly decreased (from red to beige) to allow for tensionless wound closure

**Fig. 2:**
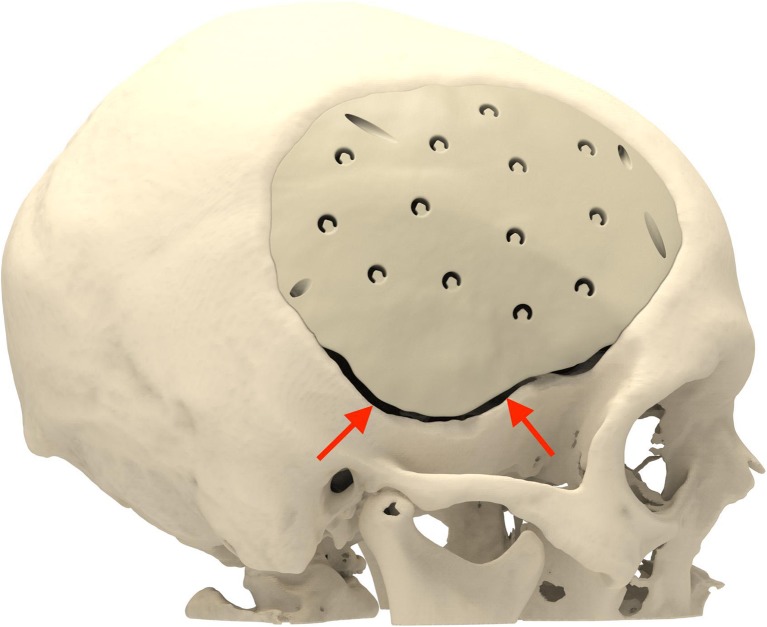
The red arrows point at the space left between the caudal edge of the implant and the bony defect edge, under the temporalis muscle (stump). Dissection of the soft tissues to find the bony rim might otherwise jeopardize the greater vessels in the area (deep temporal and middle meningeal arteries)

**Fig. 3:**
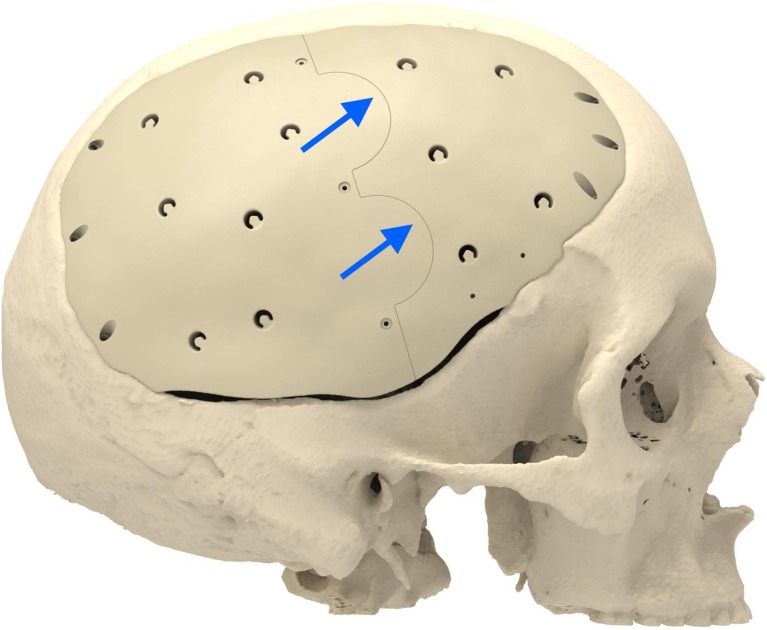
Large defects still can be covered by a PEEK implant when two segments are CNC milled and connected in situ by the way of a 3D puzzle design (blue arrows)

**Fig. 4:**
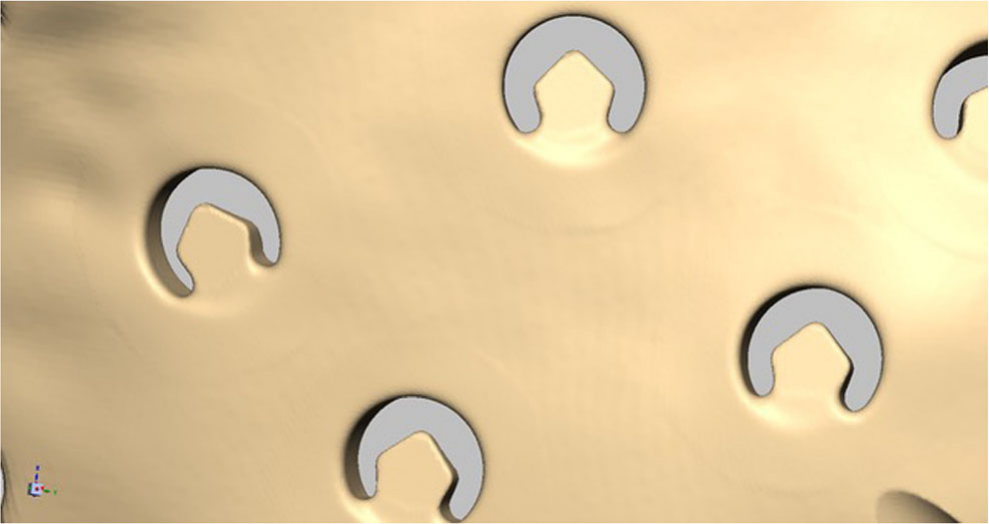
The drainage holes are at the same time available for dura and temporalis muscle suspension

**Fig. 5:**
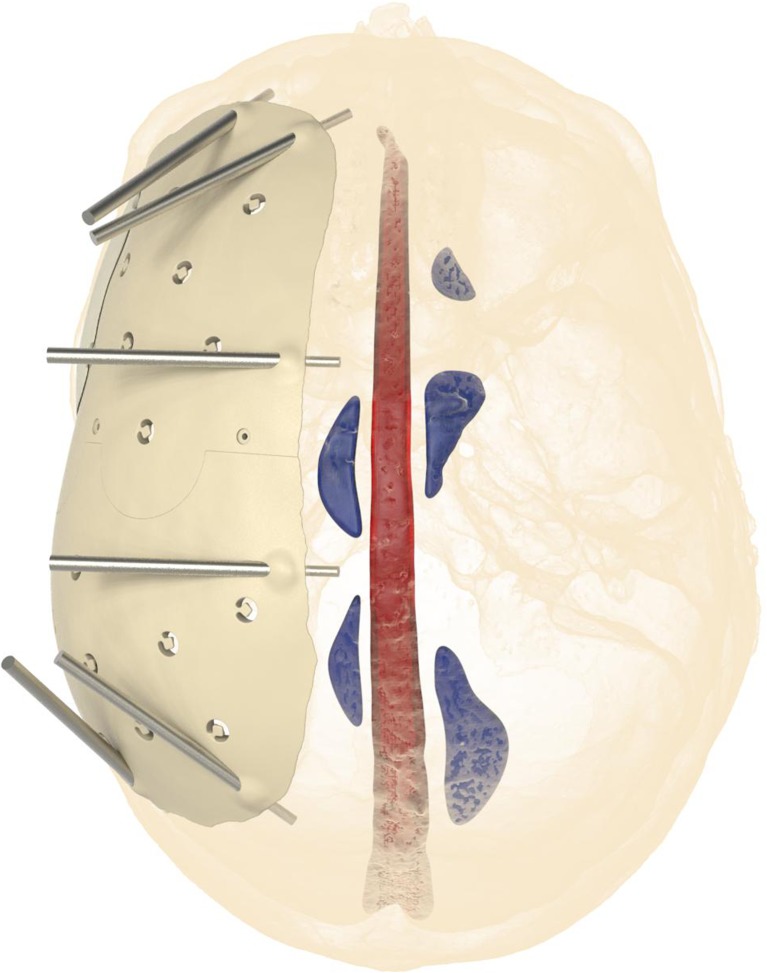
Angular holes at the perimeter are located such that drill and screwdriver access are not hindered, and the superior sagittal sinus and venous lakes are not endangered

**Fig. 6:**
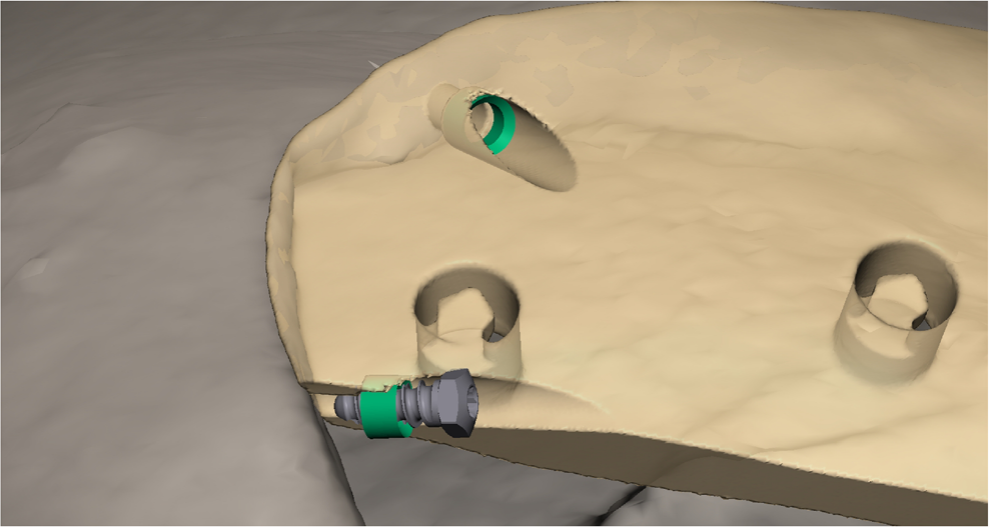
The implant border may receive too much strain from the angular fixation screw and chip off, when a biconcave washer (green) is not used to redirect the exerted force towards the screw shaft

**Fig. 7:**
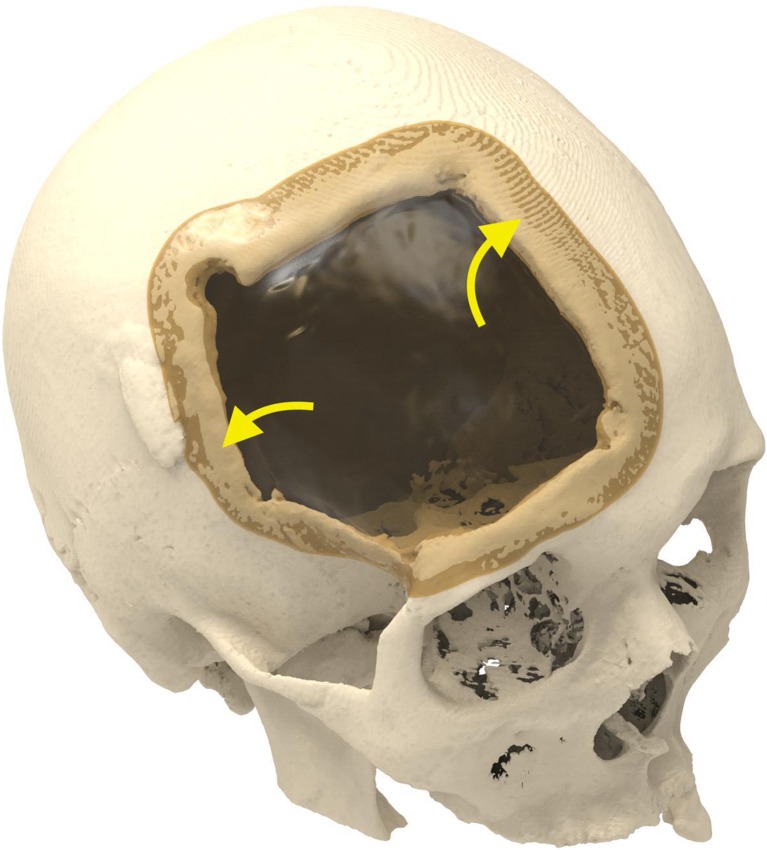
Distribution of strain upon traumatic impact by an overlapping border (yellow)

**Fig. 8:**
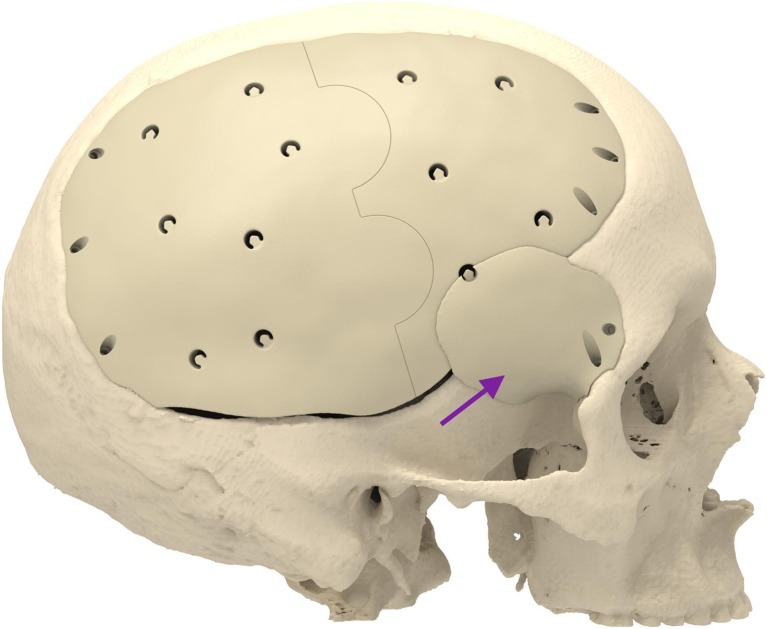
An extra implant (lila arrow) to prevent temporal hour glass deformity

Cranioplasty lends itself to customization according to the surgeon’s preferences and the patient’s condition. Revision surgery because of infection and/or resorption of the flap must take into account fibrous sheath formation and scar bands in the epidermis. Tensionless closure of the wound edges (after three or more incisions in the same area) is difficult; therefore, the contour of the implant can be slightly decreased (Fig. 1). The caudal edge of the implant descending below the pterion is shortened. Dissection of the defect perimeter under the temporalis muscle may damage the deep temporal arteries or middle meningeal arteries, resulting in difficult-to-control bleed and increased risk of epidural hematoma (Fig. 2). In extreme cases with existing large defects or profound recession/displacement of the bone flap, tissue expansion may be indicated. Large defects are more easily approached using a segmented plate, with a 3D puzzle connection for reassembly (Fig. 3), especially when PEEK is chosen as the material. PEEK implants are CNC-milled from rods that are 10, 15, or 18 cm in diameter. Two smaller plates are easier and less expensive to manufacture and produce less waste compared with a single plate, which requires a larger, thicker disk. Fluid drainage, dura suspension, and temporalis muscle suspension are facilitated by holes with interior suture lips (Fig. 4). A number of dura tenting sutures of 4–0 Vicryl (Ethicon, Johnson & Johnson, New Brunswick, NJ, USA) are threaded through the holes and secured over the lips. Angular holes at the perimeter are located such that drill and screwdriver access are not hindered by the soft tissue flap and the superior sagittal sinus and venous lakes are not endangered (Fig. 5). Such pocket hole joinery as used in carpentry (already in Ancient Egypt) found its way into orthopedic surgery (patella fracture fixation, first metacarpal fracture fixation) and into neurosurgery for fixation of cranial plates [6]. This approach also allows the plate to sink into the defect, which creates smooth bone-plate transitions. Microplate fixation should be considered for small plates in a caudal position for two reasons. First, angular holes may be difficult to approach with the drill and screwdriver when the arch segment is small and the soft tissue fold is in close proximity. Second, in small defects, the resection borders are more parallel to each other, and the implant may sink in, especially since the plates are made smaller by 0.5 mm so as to not to interfere with the boney edges. Washers are used to prevent chipping (Fig. 6) [7]. Both PEEK and titanium implants may be biofunctionalized by alumina microshot peening, acid etching, and plasma activation. The latter removes any contaminants after ultrasonic cleaning and increases wettability, thereby improving cell adhesion. Thickness can be added to the defect-filling portion so that the tapered edge overlaps the surrounding skull, which would improve the distribution of strain upon traumatic impact (Fig. 7). The temporalis muscle can be reconstructed by adding an extra shell implant of the appropriate size to be inserted only when the wound closure allows (Fig. 8).

### Patients

From June 2016 to June 2017, 18 patients underwent aforementioned cranioplasty type in the Tilburg and Brussels units. A total of 31 cranial implants were inserted, 25 made of PEEK, and 6 of titanium grade 23 ELI. The PEEK and titanium implants were fabricated by CADskills BVBA, Sint-Denijs-Westrem (Ghent), Belgium. During the study period, we gradually refined the designs as described in this report.

## Results and discussion

All implants were successful. Despite the implant material used, abnormal strain distribution in the reconstructed cranium may increase the risk of fatal trauma (Laure et al. 2010) [8]. Cranial bone has an elastic modulus of 10.4 to 19.6 GPa [1] whereas unaltered PEEK has an elastic modulus of 3 to 4, which may cause the surrounding bone to break at points where the force is concentrated (e.g., at fixation screws) [9]. Ceramic (hydroxyapatite) implants fracture more easily upon impact, causing similar problems regarding brain injury [10, 11]. Porous Ti–6Al–4V has an elastic modulus of 14.5 to 38.5 GPa and deflections that are approximately half the magnitude of those of PMMA implants [1]. In addition, stresses appear within the Ti–6Al–4V implant rather than on its perimeter, as is the case with PMMA [12], providing better protection to the brain than PEEK, PMMA, or hydroxyapatite. However, there is a concern that titanium cranioplasties may obscure details of postoperative computed tomography scans. For that reason, many will prefer a polymer implant over Ti–6Al–4V for patients with intracranial tumors. This potential problem is mitigated in patients who have skull defects from trauma or infarction [13]. Furthermore, in a series of 50 custom-made titanium cranioplasties, 4 patients received postoperative MRI and 46 patients had a postoperative CT and both imaging modalities were free of artifacts and allowed assessment of adjacent bone, meninges and brain parenchyma [14].

Titanium implants with pores of 500 to 1000 μm can facilitate the incorporation of autologous stem cells obtained from iliac crest aspirate. The interconnecting pores may allow perfusion of the overlying scalp flap but may also incorporate the dura and periosteum, making revision surgery difficult.

Plates made of polymers have the advantage that their edges can be trimmed for a better fit, avoiding the need to correct the bone borders. However, placement of polymer plates near the cerebrum is a concern because of the slow release of monomers that are known to be toxic or to cause allergic reactions. To overcome this biocompatibility problem, CNC techniques are preferred over 3D printing techniques. PEEK cages have shown excellent long-term biocompatibility in orthopedic surgery [15], and infections can be treated with intravenous antibiotics without the need to remove the infected cages. However, PEEK plates are significantly more expensive than traditional PMMA plates. A material that meets all the criteria for an ideal patient-specific skull implant is not yet available.

We found that a slight decrease in the contour of the cranial vault implant facilitates tensionless closure and wound healing without compromising the patient’s appearance. In addition, tissue expansion, which is associated with significant morbidity, may be avoided. In our view, under-contouring of the patient-specific implant should always be performed.

Despite adequate implant design, postoperative asymmetry is frequently observed in patients with temporal defects because of stripping or resection of the temporalis muscle. Initially, we used a calcium–phosphate cement to reconstruct the atrophied temporalis muscle intraoperatively. However, it was difficult to estimate the degree of atrophy, and the results were inferior. Segmenting the skull based on the subdermal outline results in a more symmetric and esthetically pleasing appearance. This technique is especially useful in cases with tissue expansion.

To overcome wound closure problems, we design the temporalis replacement piece separately. It is decided intraoperatively whether placement is indicated. When indicated and technically possible, we fix the additional plate to the vault implant using microscrews. Theoretically, the narrow space between the two PEEK plates could be prone to bacterial biofilm formation, but we have not observed any infections using this technique. A way to prevent the formation of a biofilm would be to interpose galea or temporalis muscle remnants. This extra temporal implant significantly increases the cost of the procedure.

Fixation of PEEK plates to the skull was traditionally performed using conventional maxillofacial osteosynthesis material, which required the placement of screws in the skull plates. Because osteosynthesis screws can loosen when placed in polymers, the angular fixation technique (pocket hole joinery) can be used with the advantage of pulling the skull plate into the defect. Screws or pegs can be placed between the tabulas of the calvarium. We have not observed cerebral perforations with this surgical technique.

For adequate drainage of blood and cerebrospinal fluid, we advocate the use of perforated skull plates. We designed the perforations such that they can also serve as fixation points for suspension sutures of the dura on the inner side of the skull plate and fixation points for the temporalis muscle on the outer side. We believe that suspension of the dura is essential to prevent the formation of dead space under the skull plate and facilitate cerebrospinal fluid circulation and relaxation of the brain. These tenting sutures (so-called sleeper sutures) [16] are not intended to prevent epidural hematoma formation [17]. In a retrospective review of cranioplasties using PEEK implants, O’Reilly et al. [18] found that the keys to success were meticulous closure of the skin flap, excision of the scarred galea, decreasing the dead space between the dura and implant and manufacturing the PEEK implant within 2 months after the scanning procedure.

## Conclusions

The preoperative design phase is essential to the success of the cranial implant and better esthetic outcomes. Careful design of patient-specific implants can simplify insertion and possibly diminish comorbidity by decreasing the temporal margin and using a porous structure. In addition, angular fixation can result in a more rigid fixation. Although outcomes with PEEK implants are satisfactory, there is an ongoing search to identify materials with more favorable characteristics.

## Abbreviations

CAD-CAM: Computer-aided design–computer-aided manufacturing
3D: Three-dimensional
CAD-CAM: Computer-aided design–computer-aided manufacturing
CNC: Computer numerical control
PEEK: Polyether-ether-ketone
PMMA: Poly-methyl-methacrylate
